# Mesenchymal stem cell senescence alleviates their intrinsic and seno-suppressive paracrine properties contributing to osteoarthritis development

**DOI:** 10.18632/aging.102379

**Published:** 2019-10-22

**Authors:** Olivier Malaise, Yassin Tachikart, Michael Constantinides, Marcus Mumme, Rosanna Ferreira-Lopez, Sandra Noack, Christian Krettek, Daniele Noël, Jing Wang, Christian Jorgensen, Jean-Marc Brondello

**Affiliations:** 1 IRMB, Univ Montpellier, INSERM, Montpellier, France; 2 GIGA Research, CHU of Liège and University of Liège, Liège, Belgium; 3 Clinic for Orthopedics and Traumatology, University Hospital of Basel, Basel, Switzerland; 4 Hôpital Lapeyronie, Clinical immunology and Osteoarticular Diseases Therapeutic Unit, Montpellier, France; 5 Trauma Department, Hannover Medical School, Hannover, Germany; 6 INM, Univ Montpellier, INSERM, Montpellier, France

**Keywords:** senescence, tissue homeostasis, osteoarthritis, mesenchymal stem cell

## Abstract

Tissue accumulation of p16^INK4a^-positive senescent cells is associated with age-related disorders, such as osteoarthritis (OA). These cell-cycle arrested cells affect tissue function through a specific secretory phenotype. The links between OA onset and senescence remain poorly described. Using experimental OA protocol and transgenic *Cdkn2a*^+/luc^ and *Cdkn2a*^luc/luc^ mice, we found that the senescence-driving p16^INK4a^ is a marker of the disease, expressed by the synovial tissue, but is also an actor: its somatic deletion partially protects against cartilage degeneration. We test whether by becoming senescent, the mesenchymal stromal/stem cells (MSCs), found in the synovial tissue and sub-chondral bone marrow, can contribute to OA development. We established an *in vitro* p16^INK4a^-positive senescence model on human MSCs. Upon senescence induction, their intrinsic stem cell properties are altered. When co-cultured with OA chondrocytes, senescent MSC show also a seno-suppressive properties impairment favoring tissue degeneration. To evaluate *in vivo* the effects of p16^INK4a^-senescent MSC on healthy cartilage, we rely on the SAMP8 mouse model of accelerated senescence that develops spontaneous OA. MSCs isolated from these mice expressed p16^INK4a^. Intra-articular injection in 2-month-old C57BL/6JRj male mice of SAMP8-derived MSCs was sufficient to induce articular cartilage breakdown. Our findings reveal that senescent p16^INK4a^-positive MSCs contribute to joint alteration.

## INTRODUCTION

Tissue homeostasis is ensured by the equilibrium between self-repair mechanisms of differentiated cells and their replacements through differentiation of tissue-specific adult stem cells [1]. During aging, this equilibrium is progressively lost as a result of time- dependent decrease in stem cell functions, such as self-renewal, differentiation, and tissue repair capacities [1]. Furthermore, senescent cell accumulation in tissues is one of the key processes that contribute to age-related health decline and chronic disease progression [2]. Senescent cells are characterized by growth inhibition, functional changes, and the presence of the so-called senescence-associated secretory phenotype (SASP) that includes the expression of inflammatory and trophic factors as well as tissue remodeling matrix metalloproteases (MMP) [2]. Senescence onset can occur in proliferative, post-mitotic differentiated cells, and even in resting stem cells [1, 3].

Osteoarthritis (OA), the most common osteoarticular disease, is a consequence of progressive age-induced joint senescence, leading to cartilage degeneration, osteophytosis, sub-chondral bone remodeling but also synovial hypertrophy or joint effusion [4, 5]. In conditions of homeostasis, joint cartilage relies on chondrocyte self-repair mechanisms, and on the autonomous and non-autonomous functions of the resident mesenchymal stem cells (MSCs). These non-hematopoietic CD34^-^, CD105^+^, CD90^+^, CD73^+^ cells are mainly found in sub-chondral bone marrow and the knee synovial tissue [6, 7]. *In vitro* and *in vivo* studies have shown that MSCs can form neocartilage and have remarkable tissue supportive functions through paracrine trophic factor production [8] and cell-to-cell direct contacts [9].

During OA progression and joint aging, the number of senescent cells detected in the articular cartilage, but also in the synovium and fat pad tissue, increased [10, 11]. Indeed, chondrocytes isolated from OA patients express two cell-cycle inhibitors (the senescence marker p16^INK4a^, and p57^KIP2^ [12]), and produce reactive oxygen species such as NO, remodeling catabolic enzymes but also inflammatory cytokines [11–13]. Pharmaco-genetic removal of p16^INK4a^-positive senescent cells in OA animal models demonstrated their implication in disease onset [13]. However, among all senescent cells present in the joint during OA and aging, it is not fully understood how senescence of the resident articular osteochondral progenitors (*i.e.*, MSCs) can contribute to cartilage loss of function. Therefore, in this study we aimed at determining whether p16^INK4a^-positive senescent MSCs can contribute to loss of cartilage homeostasis during OA pathogeny using *in vitro* and *in vivo* OA models.

## RESULTS

### Expression of the senescence *Cdkn2a-encoding* product p16^INK4a^ is a hallmark of experimental collagenase-induced OA and is partially required for cartilage degradation

**Figure 1:**
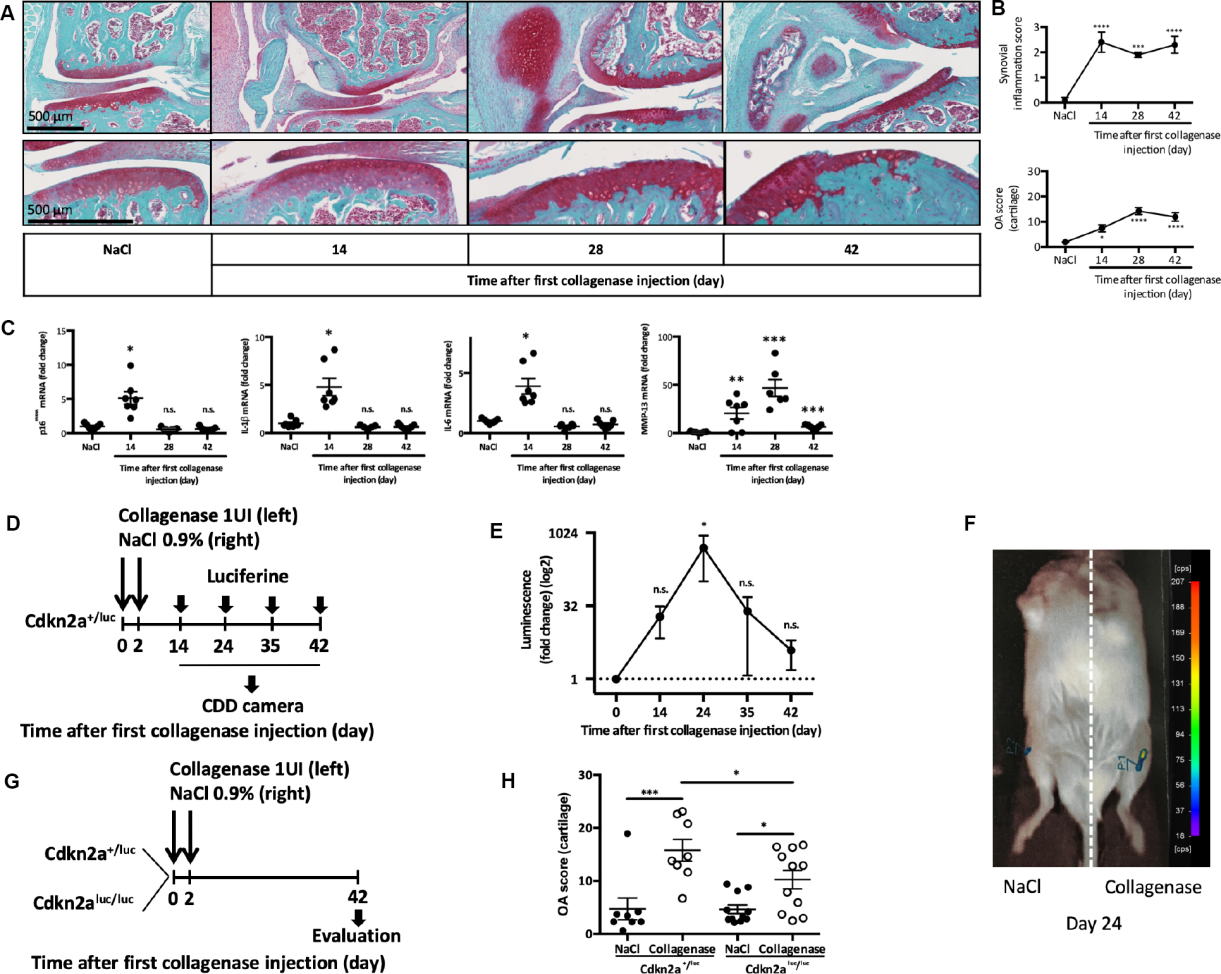
**p16^INK4a^ is involved in experimental collagen-induced osteoarthritis.** Osteoarthritis (OA) was induced by collagenase intra-articular injection in the left knee (NaCl injection in the right knee for control) of 2-month-old C57BL/6JRj male mice. (**A**) Representative images of OA kinetic development after intra-articular collagenase injection showing synovial inflammation and osteophytosis (top panel) and focus on cartilage degradation (bottom panel). (**B**) Synovial inflammation quantification (synovitis semi-quantitative score; from 0 to 3) and cartilage degradation score (OA modified score according to van den Berg; from 0 to 30) were analyzed at day 14, 28 and 42 post-injection and compared with NaCl control at day 42. Data are the mean ± SEM (n=8), *=p<0.05, ***=p<0.001, ****=p<0.0001. (**C**) p16^INK4a^, IL-1β, IL-6 and MMP-13 mRNA expression levels in the synovial membrane after NaCl or collagenase injection, measured by RT-qPCR. Results were expressed as fold change compared with NaCl control at day 42. Graphs represent the mean ± SEM (n=8); *=p<0.05, **=p<0.01, ***=p<0.001. (**D**) Experimental design of p16^INK4A^ expression analysis in *Cdkn2a*^+/luc^ after OA induction. (**E**) Luminescence analysis in both knees with a CDD camera after intra-peritoneal and intra-articular Cyc-Luc injection. Values for the left knee (collagenase injection) were expressed as fold change relative to the right knee (control). Data are the mean ± SEM (day 14, n=13; day 24, n=6; day 35, n=6; day 42 n=8); *=p<0.05. (**F**) Representative image of luciferase signal in the left (CIOA) and right (NaCl) knee at day 24. (**G**) Experimental design of OA induction in *Cdkn2a*^+/luc^ and *Cdkn2a*^luc/luc^ mice. (**H**) Cartilage degradation score at day 42 after NaCl (control) or collagenase (CIOA) injection in 2-month-old *Cdkn2a*^+/luc^ and *Cdkn2a*^luc/luc^ mice. Data the mean ± SEM (n=8 and 11 respectively); *=p<0.05, ***=p<0.001.

First, we wanted to monitor the appearance of senescent cells after OA induction in the collagenase-induced OA (CIOA) model [14], which mimics joint inflammation and synovitis that are observed in 1/3 of patients with OA [5]. To this aim, we performed intra-articular injection (at day 0 and day 2) of collagenase type VII in the left knee and saline solution in the right knee of 2-month-old C57BL/6JRj male mice*,* as previously described [15], and collected joints at day 14, 28 and 42 post-injection. Analysis of cartilage degradation (OA score) and synovitis, showed progressive cartilage degradation and early synovial activation (Figure 1A and 1B) in the collagenase-injected joint, but not the NaCl control ones.

Concomitantly, in the synovial tissue, we observed transient gene expression at day 14 of the senescence marker p16^INK4a^, but also with expression of the SASP factors (IL1-β, IL-6 and MMP13) (Figure 1C). To confirm p16^INK4a^ expression during experimental OA, we used 2-month-old *Cdkn2a*^+/luc^ mice in which the promoter of gene encoding p16^INK4a^ drives the expression of the reporter gene luciferase [16]*.* At day 14, 24, 35 and 42 after the first injection of collagenase type VII in the left knee and saline in the right knee (as before), we injected Cyc-Luc^R^ intra-peritoneally and intra-articularly, and then determined the luminescence signal intensity using a CDD camera (Figure 1D). Comparison of the two knees in each mouse showed a significant and transient peak of luciferase activity at day 24 in the OA joint following its mRNA induction at day 14 and reflecting the presence of p16^INK4a^-positive senescent cells (Figure 1E and 1F). We next asked whether p16^INK4a^ was required for cartilage and joint alteration following OA onset. To this aim, we induced CIOA in heterozygous *Cdkn2a*^+/luc^ mice that retains p16^INK4a^ protein expression and in homozygous *Cdkn2a*^luc/luc^ mice lacking p16^INK4a^ expression [16]. At day 42 post-injection, histological analysis of paraffin-embedded knee tissue sections stained with Safranin-O/Fast Green (Figure 1G, 1H and Supplementary Figure 1) showed that the mean (± SEM) OA score in collagenase-injected knees was 15.78 ± 5.8 in *Cdkn2a*^+/luc^ mice and 10.24 ± 5.8 in *Cdkn2a*^luc/luc^ mice (p<0.05). This significant difference in OA score demonstrates that p16^INK4a^ ablation partially protects against OA-driven cartilage degradation. Micro-CT analysis of histo-morphometric parameters did not reveal any difference between *Cdkn2a*^+/luc^ and *Cdkn2a*^luc/luc^ mice in term of collagenase-induced subchondral bone remodeling (bone volume, bone surface, bone surface/bone volume ratio and subchondral bone height) *(data not shown)*. This finding and the recent demonstration that specific somatic *Cdkn2a* gene inactivation in joint chondrocytes has no impact on OA onset [17] suggest that other joint cell types acquire a deleterious p16^INK4a^-driven senescence phenotype during disease development.

### Senescent p16^INK4a^-positive MSCs show impaired self-renewal and *in vitro* cartilage formation capacities altogether with specific secretory profile

Cartilage homeostasis relies primarily on the cartilage self-repair mechanisms and on MSCs found mainly in the bone marrow of sub-chondral bones and in synovial tissue [18, 19]. MSCs contribute to cartilage homeostasis through their self-renewal capacities and chondrogenic differentiation into neocartilage [20, 21]. However, MSCs might also contribute to OA onset because increased TGF-β signaling in MSCs is sufficient to induce cartilage breakdown in a transgenic mouse model [18]. Therefore, we first asked *in vitro* whether MSCs senescence could be implicated in OA pathogeny.

**Figure 2:**
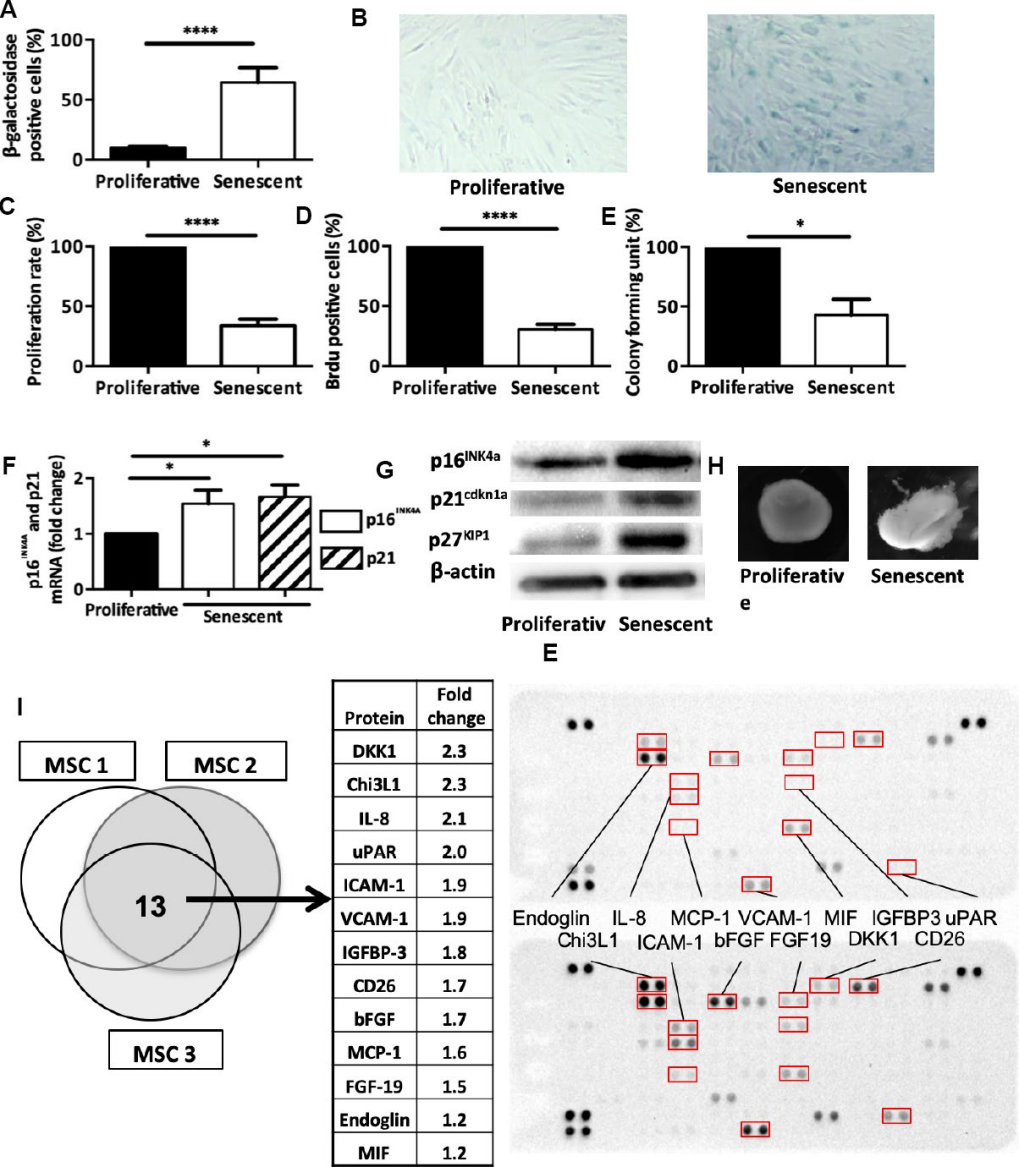
**Senescence modulates MSCs intrinsic properties.** (**A**) Beta-galactosidase staining in human MSCs at day 14 after DNA damaged-induced senescence (Senescent) or not (Proliferative). Data are the mean ± SEM (n=5); ****=p<0.0001. (**B**) Representative images of beta-galactosidase staining in proliferative and senescent human MSCs. (**C**) Proliferation rate (mean ± SEM) in proliferative and senescent human MSCs (n=6); ****=p<0.0001. (**D**) BrdU incorporation in proliferative and senescent human MSCs. BrdU-positive cells relative to all DAPI-positive cells were counted using an optical microscope (mean ± SEM; n=5). ****=p<0.0001. (**E**) Colony forming units in proliferative and senescent human MSCs (mean ± SEM; n=3); *=p<0.05. (**F**) p16^INK4a^ and p21^cdkn1a^ mRNA expression in human MSCs at day 14 after DNA damaged-induced senescence (Senescent) or not (Proliferative) by RT-qPCR. Data are the fold change relative to proliferative cells (mean ± SEM; n=4 for each condition); *=p<0.05. (**G**) p16^INK4a^, p21^cdkn1a^ and p27^KIP1^ protein expression in human MSCs at day 14 after DNA damaged-induced senescence (Senescent) or not (Proliferative) by western blotting. Representative images of MSCs from n=3 independent donors. (**H**) Representative images of one cartilage pellet after chondrogenesis induction in proliferating and senescent human MSCs (from n=3). (**I**) Protein expression profiles of total cell extracts from senescent (bottom) and proliferating (top) human MSCs from three different healthy donors. The table showed the 13 proteins that were overexpressed in all three senescent MSC samples.

To this aim, we incubated human bone marrow-derived MSCs isolated from healthy donors with etoposide, a senescence-promoting DNA-damage inducer, for 7 hours and then cultured them for 2 weeks. Following this acute treatment, MSCs showed an increase in the percentage of SA β-galactosidase-positive cells compared to untreated MSCs (64.4% ± 12.2 and 9.6% ± 3.2, respectively, p<0.0001) (Figure 2A and 2B). Proliferative rate (Figure 2C), BrdU incorporation (Figure 2D), and colony formation unit capacity (Figure 2E) were reduced in treated MSCs compared with control. Moreover, the mRNA and protein expression levels of the two main senescence markers (p16^INK4a^ and p21^cdkn1a^) were increased (Figure 2F and 2G) confirming senescence onset. We next used TGF β3-driven chondrogenic protocol to *in vitro* differentiate MSCs into cartilage. We showed that senescence induction alleviates MSC capacities to form cartilage in micro-mass setting as revealed by phase contrast imaging (Figure 2H). Finally, we performed a differential protein array analysis of senescent or proliferating MSCs allowing us to determine SASP protein expression. Among the 105 inflammation-related proteins tested, the comparative analysis identified 13 candidates that were common to three MSC isolated from healthy donors. We thus found DKK1 (a WNT antagonist factor known to trigger OA when overexpressed in a mouse model [22]), CHI3L1/YKL-40 and IGFBP-3 (two OA synovial fluid biomarkers that correlate with disease progression [23, 24]), and IL-8 and CCL2/MCP1 (two inflammatory cytokines that trigger *in vitro* senescence onset and that are usual component of the SASP [25, 26]) (Figure 2I). This profile specific of senescent MSCs by its composition could affect thus joint homeostasis by production of promoting OA and senescence spreading factors. Altogether, our findings demonstrate that following 7 hours of etoposide treatment, MSCs undergo senescence, lose their stemness properties and produce specific factors.

### Senescence alleviates *in vitro* MSCs seno-suppressive paracrine functions.

**Figure 3:**
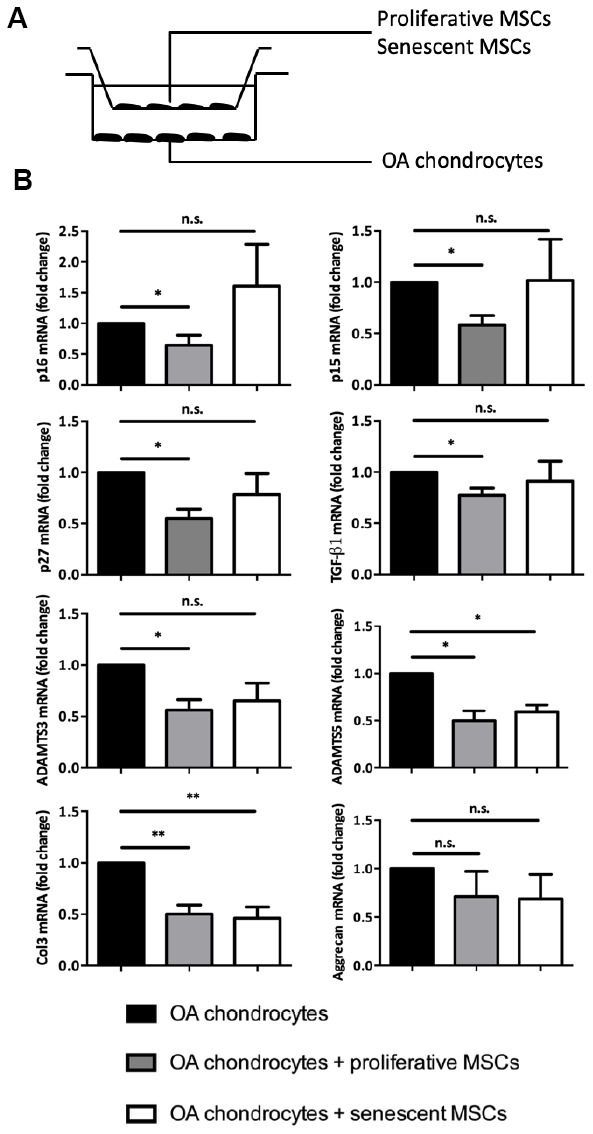
**Senescence modulates MSCs extrinsic properties *in vitro*.** (**A**) Experimental design of the without-contact co-culture system to assess the effect of senescent human MSCs on chondrocytes from patients with OA. (**B**) Expression analysis by RT-qPCR in OA chondrocytes without co-culture (black columns; control), or co-cultured with proliferating MSCs (grey columns), or with senescent MSCs (white columns) for 7 days. Data are expressed as fold change relative to control (mean ± SEM of n=5); *=p<0.05, **=p<0.01.

To maintain articular homeostasis following cartilage injury, MSC rely also on their paracrine chondroprotective properties toward functionally-altered chondrocytes. For instance, using trans-well co-culture systems, previous studies have shown that MSCs can reduce the expression of some hypertrophy and fibrotic markers in OA chondrocytes [8]. To determine whether senescence could change this central MSC tissue support function, we co-cultured for 7 days, using the same trans-well setting, OA chondrocytes with proliferative (control) or senescent p16^INK4a^-positive MSCs (Figure 3A). As previously published, control MSCs significantly reduced, in OA chondrocytes, the expression of TGF-β1, ADAMTS3 and ADAMTS5 three hypertrophic markers associated with the SASP as well as the fibrotic marker collagen 3 (Col3) (Figure 3B). Remarkably, although the secretory profile of these MSCs neither promote the re-expression of aggrecan (Figure 3B) nor collagen type 2 expression in OA chondrocytes (data not shown), it can nevertheless reduce the expression for senescence-associated cell cycle inhibitors namely p16^INK4a^, p15^INK4b^ and p27^KIP1^ (Figure 3B). Our results emphases then a chondro-protective, anti-fibrotic but also an anti-senescent paracrine effect for these stem cells towards OA chondrocytes. However, when senescent, MSCs retain only their anti-fibrotic properties by repressing Col3 *but* loss their suppressive capacities on p16^INK4a^, p15^INK4b^ p27^KIP1^ and the SASP factors TGF-β1 and ADAMTS3 in OA chondrocytes (Figure 3B). Thus, MSC seno-suppressive paracrine effect is strongly affected upon senescence onset arguing here that senescent MSC maintains and/or participates to chondrocytes loss of function during OA progression or cartilage aging.

### Senescence-accelerated SAMP8 mice develop spontaneous early osteoarthritis

**Figure 4:**
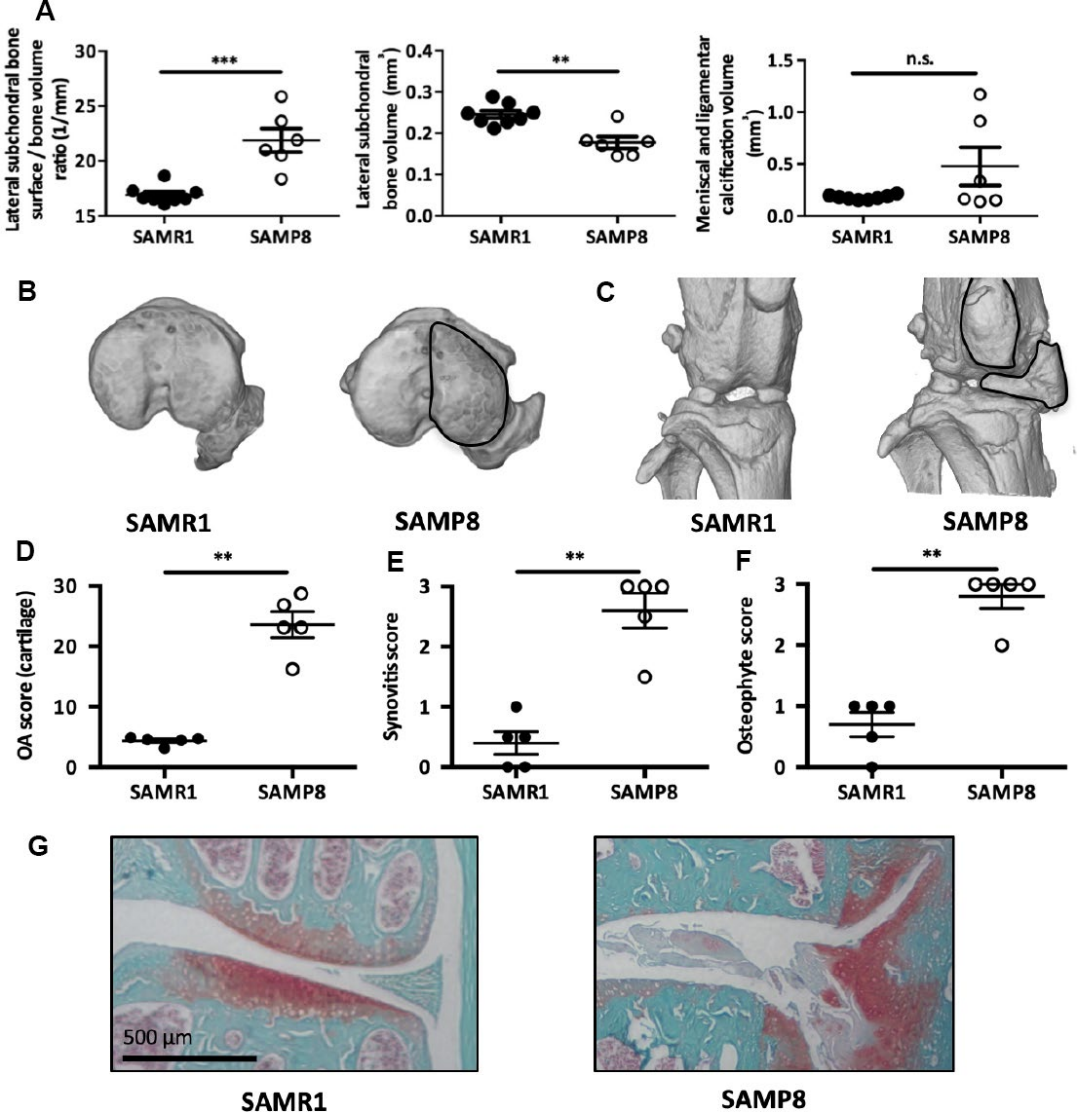
**SAMP8 mice display a spontaneous OA phenotype.** (**A**) Histo-morphometric analysis by micro-CT of the left knee in SAMR1 and SAMP8 mice. Graphs represent the mean ± SEM (n=8 for SAMR1, n=5 for SAMP8); **=p<0.01, ***=p<0.001. (**B**) Representative micro-CT images showing higher sub-chondral bone modification and (**C**) ligament calcifications in SAMP8 mice compared with SAMR1 mice. Knees from SAMR1 and SAMP8 mice were stained with Safranin-O/Fast Green to quantify: (**D**) spontaneous cartilage degradation (OA modified score according to van den Berg, from 0 to 30), (**E**) spontaneous synovial membrane inflammation (synovitis semi-quantitative score, from 0 to 3), and (**F**) osteophytes (osteophyte semi-quantitative score, from 0 to 3). Data are the mean ± SEM (n=5 for each condition). **=p<0.01. (**G**) Representative images of the spontaneous OA phenotype in SAMP8 mice with cartilage degradation compared with SAMR1 mice.

To demonstrate the causal role for senescent MSCs in OA pathogeny *in vivo*, we rely on the senescence accelerated mouse-prone (SAMP8) mouse. This strain harbors mutations in the mitochondrial genome causing an progressive increase in systemic oxidative stress, high level of cell senescence in epididymal adipose tissue [27], retinal cells [28], aorta [29], astrocytes [30], or cochlea [31], and premature aging phenotypes [27–30]. We first verify that SAMP8 prematurely develop OA. To this aim, we analyzed by micro-CT, bone and cartilage structures in 8-month-old mice revealing a significant higher bone surface/bone volume ratio and a significant lower subchondral bone volume on the lateral femoral subchondral bone area of SAMP8 compared with SAMR1 control mice (Figure 4A and 4B). This finding indicates early subchondral bone structure remodeling and degradation as found in OA patients. Ectopic ligamental and meniscal knee calcifications were also present in 50% of the analyzed SAMP8 animals (Figure 4A and 4C). Furthermore, the knee OA degradation score was significant higher in 11-month-old SAMP8 than SAMR1 control animals (Figure 4D and 4G), and was associated with higher synovitis (Figure 4E) and osteophytosis scores (Figure 4F). Altogether, this indicates that senescence-prone SAMP8 mice develop spontaneous joint OA signs that resemble the human disease.

### Intra-articular injection of senescent MSCs isolated from SAMP8 mice is sufficient to induce cartilage degeneration in young wild-type mice

**Figure 5:**
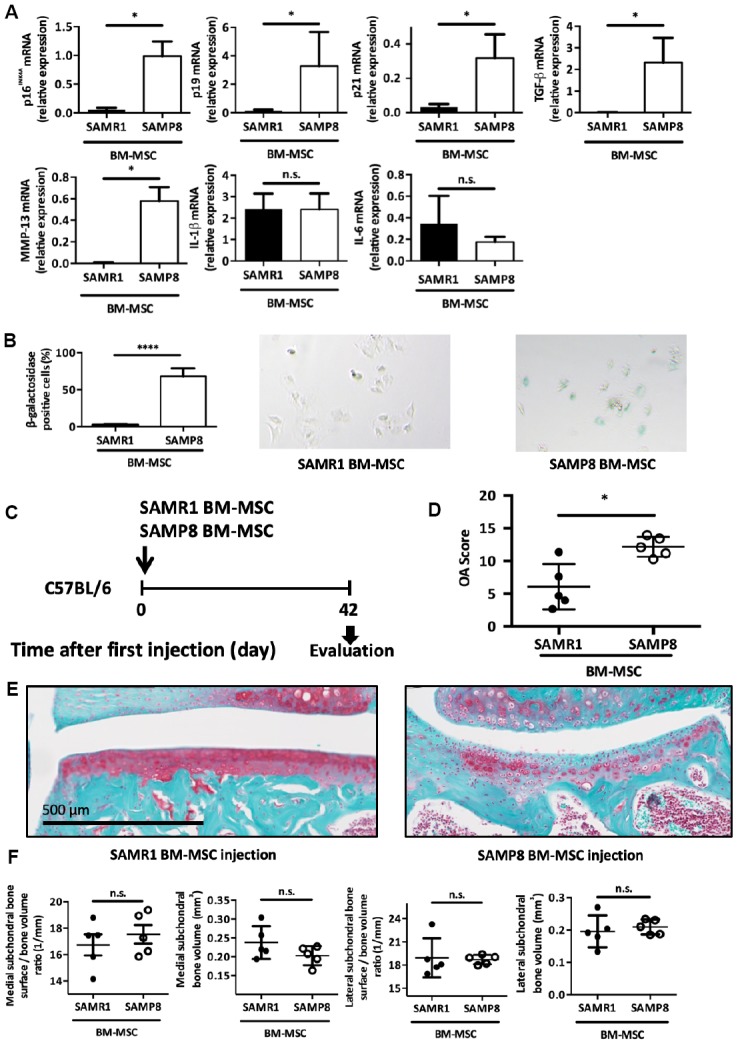
**Intra-articular injection of senescent MSCs induces OA-like cartilage degradation.** (**A**) p16^INK4A^, p19^ARF^, p21^cdkn1a^, TGF-β, MMP-13, IL-1β and IL-6 mRNA expression in MSCs derived from bone marrow (BM) of 6-month-old SAMP8 or SAMR1 mice by RT-qPCR. Data are the mean ± SEM (n=3 for each conditions); *=p<0.05. (**B**) Beta-galactosidase staining in MSCs derived from BM of SAMP8 or SAMR1 mice. Data are the mean ± SEM (n=5); ****=p<0.0001. (**C**) Experimental design of BM-MSC injection in the knee of 2-month-old C57BL/6JRj mice. (**D**) Cartilage degradation (OA modified score according to van den Berg, from 0 to 30, after Safranin-O/Fast Green staining) at day 42 after SAMP8 or SAMR1 BM-MSC intra-articular injection. Data are the mean ± SEM (n = 5 for both conditions); *=p<0.05. (**E**) Representative images of cartilage degradation in a C57BL/6JRj mouse after SAMP8 or SAMR-1 BM-MSC injection. (**F**) Histo-morphometric analyses by micro-CT of the left knee (medial and lateral compartment) at day 42 after injection of SAMR1 or SAMP8 BM-MSCs. Data are the mean ± SEM (n = 5 for each condition).

We next wanted to link premature OA development in these SAMP8 mice and the presence of senescent MSCs. To this aim, we isolated enriched mesenchymal stromal/stem population from 6-months SAMP8 and SAMR1 mice after bone marrow flushing, and 2 weeks of selection for cell culture adhesion as previously published [32]. Several cell cycle inhibitors associated with senescence state such as p16^INK4a^, p19^ARF^ and p21^Cdkn1a^, and some SASP factors namely TGF-β1 and MMP-13, were upregulated in SAMP8 MSCs compared with SAMR1 MSCs (Figure 5A) [18, 33, 34]. As expected for senescent cells, these SAMP8 MSCs showed also an increase in SA-β-galactosidase activity (Figure 5B) but a surprisingly non-classical inflammatory SASP as demonstrated by absence in IL-6 and IL-1β expression levels (Figure 5A). Then, to challenge our hypothesis whether senescent MSCs can contribute to OA development, we intra-articulary delivered 2.5x10^5^ MSCs from SAMP8 or SAMR1 mice in 2-month-old C57BL/6JRj male mice at day 0. At day 42 post-injection, each injected mouse was sacrificed and dissected joints analyzed as in Figure 4 (Figure 5C). Histological analysis after Safranin-O/Fast green staining showed that injection of SAMP8 MSCs strongly promoted cartilage degradation compared with SAMR1 MSCs (mean OA score = 12.2 ± 0.7 and 6.1 ± 1.6, respectively; p<0.05) (Figure 5D and 5E). Furthermore, subchondral bone analysis by micro-CT revealed, even if not significant, a trend toward a higher bone surface/bone volume ratio and a lower subchondral bone volume in the medial compartment in SAMP8 MSCs injected mice (Figure 5F). These results demonstrate that senescent MSCs are sufficient to trigger alone cartilage and joint dysfunction in young wild-type mice, similar to the phenotypes observed in senescence-accelerated SAMP8 mice and in human patients.

## DISCUSSION

The presence of p16^INK4a^-positive senescent cells is a well-recognized hallmark of several age-related pathologies but also ongoing tissue repair processes (for review [35, 36]). Indeed, following intrinsic and extrinsic insults, numerous tissues express senescent-induced cell cycle inhibitors such as p16^INK4a^, together with the establishment of a specific micro-environment which is normally required for tissue repair coordination [37, 38]. But during aging, secondary to their tissue accumulation, senescent cells contribute instead to the progressive loss of organ functions and to the development of age-related diseases [2].

OA is the most frequent age-related joint degenerative pathology, leading to progressive cartilage erosion and bone sclerosis [39]. Chronic low-grade inflammation and synovial hypertrophy, which is found in 1/3 of patients with OA, contribute to joint remodeling during disease progression [40]. In this work, we showed, using the collagenase-induced OA protocol to mimic articular inflammation and synovial hypertrophy [40], that joint cellular senescence transiently accumulates during disease time-course. This finding confirms recent reports based on other OA experimental-induced models showing that joint senescence is detected and contributes to OA development and cartilage aging [13, 41]. In the CIOA model, we notably observed the transient upregulation of the senescence marker p16^INK4a^ and of three SASP factors (IL1-β, IL-6 and MMP13) in the synovial tissue following collagenase injection (Figure 1). These results propose that presence of joint senescence either results from attempts to repair tissue injury or contribute to tissue degeneration.

Thus, we try to decipher which impact, negative or positive, could play p16^INK4a^-expression during OA pathogeny in the CIOA model. We then used the transgenic Cdkn2a^+/luc^ and Cdkn2a^luc/luc^ mice, in which the Cdkn2a^luc/luc^ lacks p16^INK4a^ expression. We found that after OA induction, absence of p16^INK4a^ expression significantly protects against OA-driven cartilage degradation, demonstrating rather a deleterious role for p16^INK4a^-activated cells within the injured joints (Figure 1). One knows that p16^INK4a^ regulates, through its kinase inhibitory function, the senescence-induced cell cycle arrest and also the production of articular catabolic SASP factors, giving an explanation to such effect [11, 42]. However, the observed incomplete cartilage protection in these somatic p16^INK4a^ deficient mice can be due to redundancy in other senescence signaling pathways which are therefore also implicated in the progressive cartilage breakdown [43].

Diekman et al. recently reported that inactivation of p16^INK4a^ in joint chondrocytes does not protect against OA [17]. We then wondered whether other joint resident cells can become senescent and thus could be involved in cartilage breakdown and joint loss of functions. Such cells could be tissue-resident MSCs that normally contribute to cartilage homeostasis through their stemness and articular-supportive properties. They are present in each joint compartment, especially in synovial membrane where we identified p16^INK4a^ expression. Some previous studies in mouse models suggested also that MSCs in sub-chondral bone could trigger OA onset upon increased TGFβ1 production [18]. In agreement, TGF-βR type II knock-out in MSCs prevents cartilage degradation following OA induction [18]. By becoming senescent, MSCs are therefore good candidate cells to be at the root of OA during aging.

To challenge this hypothesis, we first induced senescence *in vitro* on human primary MSC by short exposure to the DNA-damaging agent etoposide (Figure 2). This led to a reduction of MSC proliferation rate and colony formation units. Upon treatment, MSCs exhibited increased β-galactosidase activity and expression for typical cell-cycle inhibitors (p16^INK4a^, p21^Cdkn1a^ and p27^Kip1^). Furthermore, *in vitro* cartilage differentiation capacities were also impaired following senescence. Finally, protein array analysis of such MSCs revealed a senescence signature that was similar to what previously described for other cell types (*e.g.,* Chi3L1, IL8, MCP-1/CCL2, upregulation) [44] but several other proteins were specifically overexpressed by senescent MSCs. In particularly, we identified DKK1, a secreted factor normally involved in WNT signaling inhibition during hear, head and forelimb formation [45]. Of note, DKK1 level is also increased in the brain from patients with Alzheimer’s disease [45], where senescent astrocytes accumulate [46], and also in patients with osteoporosis, where senescent bone cells are found [47]. Altogether, these data and our findings suggest DKK1 as a novel senescence-associated trophic factor potentially participating to the physiological and pathological aspects of senescence during ontogeny and diseases. Although DKK1 role in OA is still unclear, several studies showed an association between high articular DKK1 levels and OA progression [48, 49]. Somatic overexpression of DKK1 in mice triggers premature bone remodeling and cartilage degradation related to OA pathogenesis [50, 51]. It would thus be interesting to evaluate in such transgenic model the presence of senescent cells in particular within the pool of resident MSC giving then an explanation how the SASP of senescent MSC leads to loss of joint homeostasis during OA and aging.

Accordingly, MSCs isolated from older donors show also an increase in senescence marker expression and a decrease in multipotency properties as in mice [47]. By creating a low inflammatory articular micro-environment, it was speculated that these MSC from elderly can favor the emergence of age-driven osteo-articular diseases [52]. Thus, it seems that senescence not only reduces the pool of functional resident articular stem cells but also their osteo-chondral capacities hence contributing to progressive joint defect with aging (Figure 2): here, we found that MSC paracrine cartilage regenerative properties are also impaired during senescence. In contrast to proliferative cells, senescent MSCs were indeed not able to reduce in OA chondrocytes, the levels of senescence and a majority of SASP/hypertrophic markers tested (Figure 3). Furthers investigations are required to determine whether this secretory phenotype in MSC leads to a gain of senescence-promoting functions through the establishment of a deleterious articular SASP (Figure 2) or on the contrary induces the loss of their seno-suppressive functions (Figures 3). Limitations for such studies reside in the absence of available healthy cartilage samples to perform proper co-culture conditions. Nevertheless, following senescence, changes in MSC interplay towards OA chondrocytes contribute to maintain joint senescence and dysfunction.

To assess whether senescent MSC can directly affect healthy cartilage functionalities *in vivo*, we injected in the joints of young mice, bone marrow stromal population isolated from senescence-accelerated SAMP8 mice. These senescent MSCs alone are sufficient to mediate OA-like cartilage degradation demonstrating *in vivo* their causative role in OA onset (Figure 5). This is reminiscent of the deleterious role of senescent MSCs isolated from SAMP6 mice, a mouse model of spontaneous osteoporosis [53] and are in agreements with the recent finding that injection of senescent human MSCs in SCID mice induces cartilage degradation [54]. Remarkably, injected senescent MSCs were extracted from SAMP8 mice suffering from a spontaneous OA cartilage phenotype, synovial inflammation, osteophytosis and subchondral bone modification (Figure 4). This spontaneous osteoarthritic condition makes SAMP8 strain mouse as an interesting model for senescence-driving OA studies. All these dysfunctional osteo-articular tissues revealed in SAMP8 rely normally on MSC for homeostatic maintenance. Finding senescence markers in SAMP8-derived MSCs gave an overview on the impact of senescent stem cells on joint functionality. Accordingly, senescent SAMP8 MSCs when injected into young mice can also trigger such pleiotropic osteo-articular dysfunctional phenotypes. This finding confirms our hypothesis that MSC senescence can have axial deleterious articular functions in some pathological conditions.

It was also shown by others that senescent MSCs can alter the subchondral bone micro-environment and structure by increasing osteoclast differentiation and adipocyte differentiation [55]. We can therefore conclude that cumulative senescence in MSCs will affect all joint compartments, contributing to OA development, progressive bone restructuration and articular aging. This seems to be a global pro-aging driving phenomenon common to other mesodermal tissues. For instance, senescent MSCs are observed in myelodysplasic syndrome [56], accumulate in the nucleus pulposus of the intervertebral degenerative disc in older patients [57], and senescent MSCs are found early in Hutchinson Gilford Progeria syndrome [58]. Altogether, our findings raise the need to identify seno-therapeutic agents that specifically target senescent MSCs with the aim of improving the regenerative potential of all osteo-articular tissues in elderly [44].

## MATERIALS AND METHODS

### Animal experiments

6-weeks-old C57BL/6JCj male mice were obtained from Janvier Laboratory. SAMP8 and SAMR1 mice were bought from Envigo laboratory. Transgenic *Cdkn2a*^+/luc^ and *Cdkn2a*^luc/luc^ mice were obtained from K.B. Sharpless’s laboratory. Animal experiments were performed in accordance with the guidelines by the local ethics committee on animal research and care (approval CEEA-LR-10042). After sacrifice, soft tissues and muscles around the knee were dissected and removed. Patella was removed and the supra-patellar synovium tissue was isolated and stored in 200μL Trizol at -80°C. The knee joint was separated, fixed in 4% paraformaldehyde at 4°C for 2 days, and decalcified by incubation at +4°C in a large volume of 14% EDTA for 21 days. The EDTA solution was changed every 3 days. Samples were then stored in PBS at +4°C.

### Experimental osteoarthritis and intra-articular injections

Experimental OA was induced in 6-weeks-old male mice according to the CIOA procedure. Under general anesthesia (isoflurane inhalation), the knee area was disinfected with ethanol and a small cut (a few millimeters) was performed in the cutaneous and subcutaneous tissue to visualize and to access the knee. Then, each mouse received an intra-articular injection of 5μL (1IU) collagenase VII in the left knee to induce OA and 0.9% NaCl in the right knee as control, followed by a second injection two days later. Intra-articular injections of BM-MSCs were performed in 6-weeks-old male mice following the same procedure: 8μL of PBS containing 2.5x10^5^ BM-MSCs isolated from SAMP8 or from SAMR1 mice in the left knee at day 0.

### Bioluminescence imaging

For luciferase signal detection, mice were anaesthetized before intraperitoneal (100μL) and intraarticular (10μL) injection of the luciferase substrate Cyc-Luc (300μg/mL) (Merck). A few minutes later, mice were placed in the dark chamber and a grey-scale image was first recorded with dimmed light followed by acquisition of luminescence images using a cooled charged-coupled device (CCD) camera (PIXIS 1024B; Princeton Instruments).

### Bone parameter analyses and Safranin-O/Fast Green staining for cartilage analysis

Hind leg paws were dissected to remove smooth tissues and scanned in a micro-CT scanner SkyScan 1176 (Bruker, Belgium, 0.5 mm aluminum filter, 45 kV, 500 μA, 18 μm resolution, 0.5° rotation angle). Scans were reconstructed using CTAn v1.9, Nrecon v1.6 (Bruker, Belgium) and a three-dimensional (3D) model visualization software program (CTVol v2.0). Misalignment compensation, ring artifacts and beam-hardening were adjusted to obtain the correct reconstruction of each paw. Bone degradation was quantified in subchondral bone and the epiphysis region of the medial and/or lateral plateau for each tibia (CTAn software, Bruker, Belgium). Meniscal and ligament calcifications were quantified on the entire knee joint. Reconstructed 3D images of joints were obtained using the Avizo software (Avizo Lite 9.3.0, FEI, France). For cartilage analysis, mouse knees were fixed in 4% paraformaldehyde at 4°C for 48h, washed in PBS, and then processed for routine histology. Knees were decalcified in 14% EDTA/PBS for three weeks and then paraffin-embedded. Tissue sections (5 μm) were rehydrated through a gradient of ethanol and xylene. Sections were then stained with Safranin-O/Fast Green solution to evaluate cartilage degradation, synovial inflammation and osteophytosis. Cartilage damage was analyzed using an arbitrary score of 0–30, based on the OARSI cartilage OA grading system histopathology [59], and modified by van den Berg for the assessment of murine knee joints (grading scale of 0–6 for the severity of cartilage destruction and of 0–5 for the extent of damaged cartilage surface) [14, 60]. A semi-quantitative scoring system was used to analyze synovitis, with scores ranged from 0, indicating no thickening of the lining layer (1 cell layer), to 3, indicating the maximal observed thickening of the synovial lining layer [14, 60]. The same semi-quantitative scoring scale, from 0 to 3, was used for osteophytosis, with 0 = no osteophytosis and 3 = the largest osteophytosis observed [61, 62].

### Cell types and culture conditions

Human MSC cultures were established from bone marrow aspirates of healthy donors after signature of the informed written consent and approval by the local and national ethics committees (committee of Hannover Medical school with votum No. 2562). MSCs were isolated and amplified in complete alpha-minimum essential medium (Ozyme; BE12-169F-12) supplemented with 10% fetal bovine serum (FBS), 1% penicillin/streptomycin, 2 mM L-glutamine, and 1 ng/mL of basic fibroblast growth factor-2 (Miltenyi Biotec; GER 130-104-924). For DNA damage-induced senescence, MSCs were incubated with 12.5 μM etoposide (E1383 Sigma, USA) for 7h. Senescence levels were evaluated after 14 days (medium changed every 3 days). Human OA chondrocytes were isolated from cartilage of patients with OA undergoing knee arthroplasty after signature of the informed written consent and approval by the national ethics committee (‘Cellule de bioéthique de la direction générale pour la recherche et innovation, Ministère de l’Enseignement Supérieur et de la Recherche’; registration number DC-2009-1052). OA chondrocytes were cultured in Dulbecco’s modified Eagle’s medium (DMEM) containing 10% fetal calf serum (Sigma, USA), 1% penicillin/streptomycin (Lonza, CH), and 2 mM L-glutamine (Lonza, CH).

### Bone marrow mesenchymal stem cell isolation

Bone-marrow MSCs (BM-MSC) were isolated from the right leg of SAMP8 and SAMR1 mice. Femur and tibia were dissected, and diaphyses were flushed with PBS. Cell suspensions were filtered and plated at a concentration of 1x10^6^ cells/cm^2^ in DMEM medium supplemented with 10% FBS (Hyclone, Thermo Fisher Scientific), 2mM glutamine, 100U/mL penicillin, 100mg/mL streptomycin (Lonza, Levallois-Perret, France) and 2ng/mL human basic fibroblast growth factor (R&D Systems, Lille, France). Medium was changed daily to remove red blood cells and non-adherent cells. BM-MSCs were stored at -80°C after passage 1.

### Co-culture experiments

Co-culture experiments were performed as previously described [8]. Briefly, 5x10^5^ OA chondrocytes/well were plated on the bottom of 6-well plates and cultured with MSCs (7x10^4^ cells/insert) in cell culture inserts (PET membranes, 0.4 μm pores, BD Biosciences, UK). Cultures were maintained in 3 mL of minimal medium (DMEM supplemented with 100U/mL penicillin and 100mg/mL streptomycin, 0.35 mmol/L proline, 0.17 mmol/L ascorbic acid, and 1 mmol/L sodium pyruvate) for 7 days when chondrocytes were collected for RT-qPCR analysis.

### Gene expression analysis by real-time quantitative PCR

For qPCR experiments, total RNA was extracted from cells using the RNeasy® Mini kit (Qiagen; 74106) according to the manufacturer's instructions. RNA quality was checked by spectral analysis (A260/ 280 nm), and then samples were stored at −80°C. Reverse transcription was performed using the M-MLV reverse transcriptase (Invitrogen; 28025013; 5U/μL final concentration), 500ng total RNA, a random hexamer primer (Thermo Scientific, GER; S0142; 10ng/μL final concentration), and DNTPs (Roche, CH; 1 277 049; 5mM final concentration) in M-MLV reverse transcriptase buffer (Invitrogen; 18057-018), for a total volume of 20μL. SYBR Green-based quantitative PCR was performed using the LightCycle® 480 SYBR Green I master reaction mix (Roche, 04707516001), 10 ng of cDNA, and the LightCycler 480 real-time PCR system (Roche) (40 cycles of amplification). Raw data (Ct values) were analyzed using the comparative Ct method. Gene expression data were calculated as relative to the expression of housekeeping genes. The comparative threshold cycle method (ΔΔCT) was used to quantify relative gene expression, and the obtained quantification was transformed to exponential 2−ΔΔCT values. A p-value below 0.05 (Student’s *t*-test) was considered as statistically significant. Primers sequences used for RT-qPCR are listed in Supplementary Table 1.

### Proliferation, Colony Forming Unit (CFU) assays and BrdU incorporation

For CFU assays, 3x10^3^ MSCs were homogeneously plated in 60mm dishes. After 10 days, when cell colonies emerged, cultures were fixed with methanol and stained with Giemsa dye solution diluted 20X for counting. For BrdU incorporation analysis, 12x10^3^ MSCs were plated on coverslips 14 days after etoposide treatment or not. Cells were incubated with 10μM of BrdU in fresh culture medium for 21 hours, and then fixed with 3.7% paraformaldehyde solution at room temperature (RT) for 15min. Cells were permeabilized by adding 0.3% Triton X-100 in PBS at RT for 30min. After Triton X-100 removal, 500μL of 2N HCl solution was added at RT for 10min, followed by incubation with 10% donkey serum in PBS at RT for 30 min. Then, cells were incubated with rat anti-BrdU IgG (1:250 in 3% BSA/PBS, Abcam; ab6326) at 37°C for 2 hours, followed by 488 Alexa Fluor® conjugated goat anti-rat IgG (1:500 in PBS, Thermo Fisher Scientific; A-11006) at RT for 1 hour. Coverslips were mounted with Prolong Gold Antifade Reagent (Thermo Fisher Scientific; 11569306) containing 4′-6-diamidino-2-phenylindole (DAPI). Cells with nuclear BrdU signal were counted as positive among at least 100 DAPI-positive cells from 10 images of randomly chosen fields for each sample, using the ImageJ software.

### *In vitro* chondrogenic differentiation

Chondrogenic differentiation of MSCs was induced by 21-day culture in micropellet, as explained previously [63]. Briefly, MSCs (2.5 × 10^5^ cells) were pelleted by centrifugation in 15 ml conical tubes and cultured in chondrogenic medium (DMEM supplemented with 0.1 μM dexamethasone, 0.17 mM ascorbic acid, 1% insulin-transferrin-selenic acid (ITS), 350 nM proline, 1 mM sodium pyruvate) supplemented with 20 ng/mL TGFβ-3.

### Senescence-associated β-galactosidase activity

Induction of MSC senescence was assessed by staining MSCs with a senescence-associated β-galactosidase (SA-β-Gal) Staining Kit (Sigma-Aldrich; CS0030) according to the manufacturer's instructions. In detail, 12x103 MSCs were plated on coverslips and after 24h, they were fixed with the Kit fixation solution at RT for 8min, and then they were incubated with the SA-β-Gal staining solution overnight to reveal SA-β-Gal activity. Cells displaying blue signal in the cytosol were counted as positive, using the ImageJ software. Ten images of random fields for each sample were taken by microscopy, and at least 100 cells were counted for each sample.

### Inflammation protein profile analysis and western blotting

The secretome of senescent and proliferating MSCs was analyzed with the Protome Profiler Human XL Cytokine Array Kit (R&D Systems; ARY022B) according to the manufacturer’s instructions for the parallel determination of the relative level of 105 human cytokines, chemokines, and acute phase protein. Capture antibodies were spotted in duplicate on nitrocellulose membranes to bind to specific target proteins present in the cell supernatant. Captured proteins were detected with biotinylated antibodies and visualized using chemiluminescence detection reagents. For western blot analysis, proteins were isolated from cultured MSCs using Lysis buffer 17 (R&D systems; 895943), supplemented with protease inhibitors (Roche; 11 873 580 001), according to the manufacturer's instructions. Protein concentration was estimated using the BCA Protein assay (Fisher Scientific; 10678484) and 50μg of total proteins was loaded per well after denaturation in Laemmli blue with beta-mercaptoethanol (at 95°C, for 7min). Samples were resolved through Mini-PROTEAN® TGX™ 4-15% gradient gels (Bio-Rad: 456-1084) in Tris-glycerin buffer, and then were transferred to polyvinylidene fluoride (PVDF) membranes (Bio-Rad Transblot® Turbo™: 170-4157) using the Bio-Rad Transblot® Turbo™ system (1.3A – 25V – 7 min). After transfer, membranes were washed in Tris-buffered saline (TBS), blocked in 5% non-fat dry milk in TBS-0.1% Tween 20 (TBST) at RT for 1 hour, followed by incubation with primary antibodies at 4°C overnight, except for the anti-β-actin antibody (RT for 3h). The following primary antibodies were used: rabbit IgG anti-human p16 (1:750 in 5% NFDM-TBST, Proteintech; 10883-1-AP), rabbit IgG anti-human p21 (1:1000 in 3% BSA-TBST, Abcam; 109199), mouse IgG anti-human p27 (1:200 in 5% NFDM-TBST, Abcam; 193379), rabbit anti human β-actin (1:1000 in 5% BSA-TBST, Abcam; ab8226). After incubation, membranes were washed in TBST and incubated with goat anti-rabbit IgG (1:2000 in 5% BSA-TBST; Cell Signaling, 7074) or goat anti-mouse IgG (1:2000 in 5% BSA-TBST, Cell Signaling; 7076) horseradish peroxidase (HRP)-conjugated at RT for 2h. Reactions were visualized using the Immobilon ECL HRP Substrate (Millipore; WBLS0500).

### Statistical analysis

All data are presented as the median or mean ±SEM or SD. The Student’s *t*-test was used for comparisons between experimental groups, and ANOVA for multiple comparisons followed by a Sidak post-test. Data were analyzed using the Prism software v6 (GraphPad Software Inc.); p-values <0.05 were considered significant (*p <0.05; **p <0.01; ***p <0.001; ****p <0.0001).

## Supplementary Material

Supplementary Figure 1

Supplementary Table 1
