# Supplementary material for: Mesenchymal stem cell senescence alleviates their intrinsic and seno-suppressive paracrine properties contributing to osteoarthritis development
Source: Aging (Albany NY). 2019 Oct 22;11(20):9128–46. doi: 10.18632/aging.102379 (PMC6834426; doi:10.18632/aging.102379)
Supplement: Supplementary Figure 1 [file aging-11-102379-s001.pdf]

SUPPLEMENTARY FIGURE

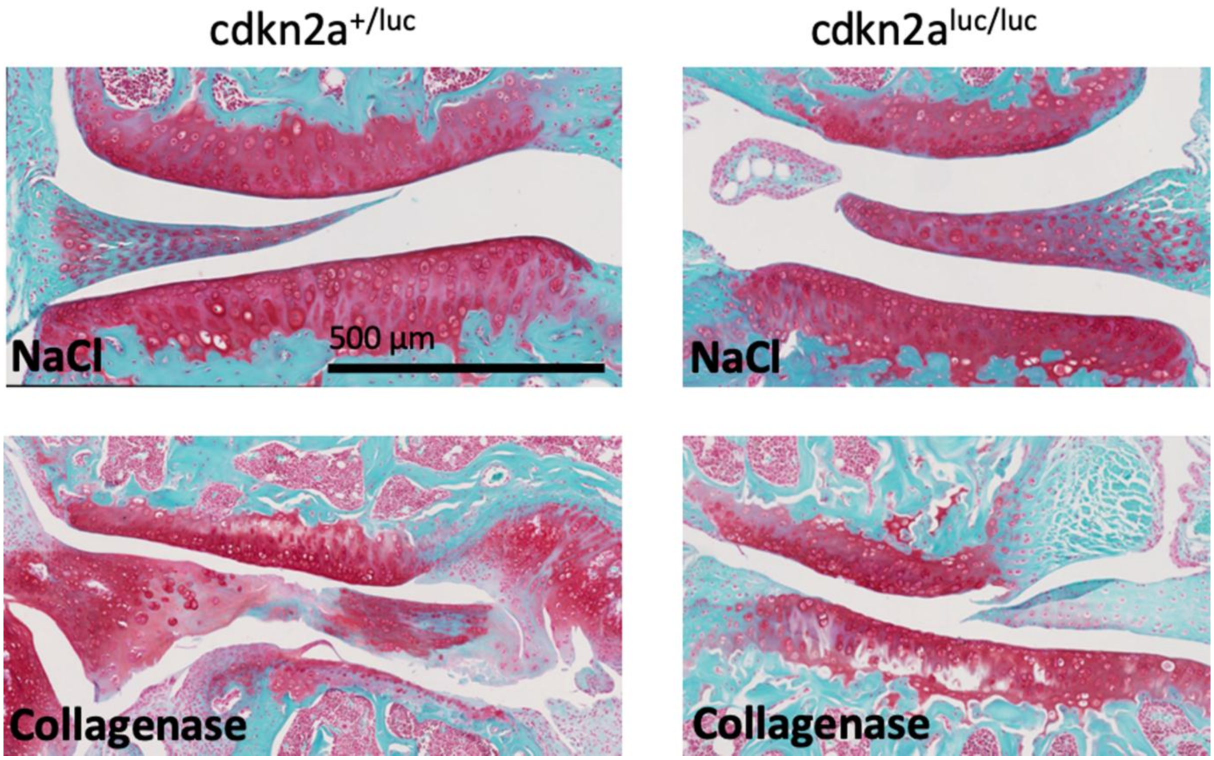

Supplementary Figure 1. Representative images of cartilage degradation at day 42 after NaCl (control) or collagenase (CIOA) injection in 2-month-old *Cdkn2a*$^{+/luc}$ and *Cdkn2a*$^{luc/luc}$ mice.
