# Supplementary material for: Mesenchymal stem cell senescence alleviates their intrinsic and seno-suppressive paracrine properties contributing to osteoarthritis development
Source: Aging (Albany NY). 2019 Oct 22;11(20):9128–46. doi: 10.18632/aging.102379 (PMC6834426; doi:10.18632/aging.102379)
Supplement: Supplementary Table 1 [file aging-11-102379-s002.pdf]

SUPPLEMENTARY TABLE

Supplementary Table 1. Primer list.

Gene (human)	Forward primer 5′-3′	Reverse primer 5'-3′
p16^{INK4a}	GAAGGTCCCTCAGACATCCCC	CCCTGTAGGACCTTCGGTGAC
p21^{cdkn1a}	TGTCCGTCAGAACCCATGC	AAAGTCGAAGTTCCATCGCTC
p15^{INK4b}	GACCGGGAATAACCTTCCAT	CACCAGGTCCAGTCAAGGAT
p27^{KIP1}	CGGCTAACTCTGAGGACACG	CTTCTGAGGCCAGGCTTCTT
TGF-β1	CTAATGGTGGAAACCCACAACG	TATCGCCAGGAATTGTTGCTG
ADAMTS3	GAACATCGACCAACTCTACTCCG	CAATGCCCACCGAACCATCT
Aggrecan	TCGAGGACAGCGAGGCC	TCGAGGGTGTAGCGTGTAGAGA
Col3	AGGGCCTGA AGGACCAGCTT	CGCCCTCCTAATGGTCAAGG
28S	CGATCCATCATCCGCAATG	AGCCAAGCTCAGCGCAAC
**Gene (mouse)**	**Forward primer 5′-3′**	**Reverse primer 5′-3′**
p16^{INK4a}	CGCAGGTTCTTCGTCACTGT	TGTTCACGAAAGCCAGAGCG
p19^{ARF}	GCTCTGGCTTTCGTGAACATG	TCGAATCTGCACCGTAGTTGAG
p21^{cdkn1a}	GTACTTCCTCTGCCCTGCTG	TCTGCGCTTGGAGTGATAGA
IL-1β	GTATGGGCTGGACTGTTTC	GCTGTCTGCTCATTCACG
IL-6	TAGTCCTTCCTACCCCAATTTCC	TTGGTCCTTAGCCACTCCTTC
MMP-13	GGAGCCCTGATGTTTCCCAT	GTCTTCATCGCCTGGACCATA
GAPDH	GGCAAATTCAACGGCACAGT	GTCTCGCTCCTGGAAGATGG
